# Some Light on a Recent Controversy

**Published:** 1921-07-09

**Authors:** 


### TONSILS AND ADENOIDS.

### Some Light on a Recent Controversy.

It is a curious fact that although enlargement of the tonsils and adenoids in children is such a common disease, the etiology is still largely unknown, and this situation probably accounts for the repeated appearance of claims for non-operative methods of cure. Some of these have been obviously absurd. Others are merely misrepresentations of what can be expected from rational and hygienic living. Such performances as "nasal drill" can, and undoubtedly do, improve the general condition of a child, reduce the local catarrh, and indirectly aid normal development, but it is more than doubtful whether thereby any actual reduction of the lymphoid hypertrophy can be effected. Whichever evidence we take, the fact remains that the quickest and most certain method of cure is surgical, unless the child is for some reason unfit to withstand the procedure.

### An Inquiry.

This question brings us, however, to the recent outcry raised by a well-meaning and in most ways most excellent society for a complete inquiry into the tonsils and adenoids work in our hospitals. To some extent and in some places such a review might be advantageous, but, as with all such controversies between the layman and the profession, there are points in the latter's argument which it is exceedingly difficult for the former to understand. For years the removal of tonsils and adenoids has been carried out under out-patient clinic conditions. A surgeon experienced in this work will describe thousands of perfectly successful cases, operated upon so easily and so quickly that the use of a general anæsthesia in the accepted sense is really unnecessary. The children in these circumstances do not suffer either acute pain or severe hæmorrhage, and the post-operative treatment of gargles, etc., can be carried out at home with entirely satisfactory results. The experienced surgeon does not choose unsuitable cases, he sees and examines them all before the actual day of operation, and again he sees them before they go home after it. The risk and percentage of unsatisfactory results in our big hospitals is absurdly small to have created so great an outcry, and the surgeons on their staffs are rightfully indignant.

But there is another side to be considered, the practical as against the professional side. The hospital, waiting-lists for tonsils and adenoids are of ever-increasing length, both in this and other countries, and correspondingly the necessity grows that the expert surgeon shall call upon his less experienced assistants. More cases must be done in the same time, and gradually it becomes less possible for close individual attention to be given to each. Then, small though it still is, the risk inevitably increases, and one begins to sympathise, not with those who would abolish the out-patient clinics for tonsils and adenoids, but with those who would insist on precautions to counteract the danger.

One of the most recent surveys of this whole problem has been carried out by the Public Health Committee of the New York Academy of Medicine. The report, as published in a recent number of the *Medical Record*, gives some useful facts and figures for comparison with our own. It is found that in the ten years ending 1919 true tonsil and adenoid defects occurred in 10 to 19 per cent. of the children examined, and the later years showed a considerable relative increase. The large majority of these notified children were considered as definitely needing operative treatment, giving an annual demand for some 55,000 operations, whereas the actual number treated surgically in 1920 fell some 8,000 below this demand.

### The Example of New York.

On this account a review of the possibilities in New York has been made, and the first conclusion arrived at is that a more uniform distribution of the patients *must* be made. It would seem that the established reputation and popularity of certain hospitals are partly responsible for the long waiting-lists, and it is proposed to establish a bureau for advice and guidance as to where facilities are available at any given time. The standards of operative treatment, as well as of care of the patients, differ considerably in the several hospitals. In many, for example, it is found that a more complete physical examination of the patients might be made before operation, and there should be more effective methods of instruction as to home care of the children when they leave hospital. The Committee (1) state that only a few dispensaries are now attempting tonsil operations; (2) remind us that the Academy's Section of Laryngology and Rhinology declares that tonsillectomies should not be attempted at out-patient clinics; and (3) quote a re-examination of a series of treated children, satisfactorily proving to them (the Committee) that without anæsthetics a large number of operations are incompletely performed.

Therefore they urge that not only should some inter-hospital system be developed whereby the institutional facilities can be brought to their maximum effect, but also that all tonsils and adenoids operations on children should be done under general anæsthesia, that hospitals keep the patients for a correspondingly longer time, and take more thorough interest both in selection and after-care of their patients.

To many of us all this will seem to suggest an exaggerated importance given to a very trivial operation, but from the national standpoint the matter is well worth consideration. Those with the care of our children are well advised to urge to the full all the established preventive measures against development of the adenoid state. Whatever success they achieve is all to the good.. But the need for carefully organised surgical treatment will remain with us, as with New York, for many a day.

